# Association between Pain and Frequent Physical Exercise among Adults in the United States: A Cross-Sectional Database Study

**DOI:** 10.3390/sports11070126

**Published:** 2023-06-29

**Authors:** David R. Axon, Taylor Maldonado

**Affiliations:** 1 Department of Pharmacy Practice & Science, R. Ken Coit College of Pharmacy, The University of Arizona, 1295 N. Martin Ave., Tucson, AZ 85721, USA; tamaldonado0812@arizona.edu; 2 Center for Health Outcomes and Pharmaco Economic Research (HOPE Center), R. Ken Coit College of Pharmacy, The University of Arizona, 1295 N. Martin Ave., Tucson, AZ 85721, USA

**Keywords:** physical exercise, pain, United States adults

## Abstract

Pain affects over 20% of United States adults, and less than 50% of United States adults participate in frequent physical exercise. This cross-sectional database study included 13,758 United States adults aged >18 years from the 2020 Medical Expenditure Panel Survey (MEPS) and analyzed the association between severity of pain (independent variable) and frequent physical exercise (dependent variable), adjusting for demographic, economic, limitation, and health variables using multivariable logistic regression. The study showed 50.3% of adults report frequently exercising. Only 37.1% of adults reported experiencing pain of any degree, with a majority of them experiencing little pain. In the adjusted model, extreme pain vs. none, quite a bit of pain vs. none, Hispanic vs. non-Hispanic ethnicity, having a functional limitation vs. no limitation, and being overweight/obese vs. not being obese/overweight were associated with lower odds of reporting doing frequent physical exercise. Meanwhile, being ≥65 or 40–64 vs. 18–39 years of age, male vs. female, white vs. not white race, private or public vs. no health coverage, and good vs. poor general health were associated with greater odds of reporting doing frequent physical exercise. These variables associated with frequent physical exercise should be considered in future work when designing health interventions.

## 1. Introduction

Pain may be defined in a number of ways, but one common descriptor offered by the International Association for the Study of Pain is “an unpleasant sensory and emotional experience associated with, or resembling that associated with, actual or potential tissue damage” [1]. Over fifty million adults experience pain every day, indicating one in five adults in the United States (US) experience pain. Of those adults, 79% experience pain in their back; 68% experience pain in their hands, arms, or shoulders; 79% experience pain in their hips, knees, or feet; 42% experience headache or migraine; 23% experience pain in their abdomen, pelvis, or genitals; and 21% experience pain in their teeth or jaw [2].

Moderate to high-intensity workouts improve physical function without worsening feelings of pain, as the pain comes from chronic cellular inflammation which can be mitigated by physical activity. Aerobic exercise is one of the best ways to increase mobility and decrease pain in day-to-day tasks [3]. Aerobic exercise involves continuous movement utilizing all major muscle groups to strengthen the cardiothoracic region. For the heart to grow stronger, aerobic exercise should last continuously for 20–60 min three to four times a week [4]. Different examples of aerobic exercise include swimming, cycling, walking, rowing, running, and high intensity interval training [5]. The Department of Health and Human Services recommends adults should be exercising anywhere from 150 min to 300 min a week with moderate intensity or 75 min to 150 min a week with intense aerobic activity to maintain and improve health [6]. Frequent physical exercise can improve brain health, manage weight, reduce the risk of disease, and strengthen bones and muscles [7]. These benefits, especially those regarding brain health, can be seen immediately after moderate exercise [7].

Pain is often self-managed by individuals who use a variety of different approaches [8,9]. Interdisciplinary treatment is important for pain management, which can include self-care, nutrition, exercise, and smoking cessation [8,9,10,11]. The association between pain and physical exercise is an emerging research area that is not yet completely understood [12]. However, it is known that sedentary behavior may lead to increased pain sensitivity [13]. For instance, Naugle et al. used a conditioned pain modulation test to show that people who were less sedentary felt less of the heat directed at their bodies, implying that they would feel less pain [14]. Studies have also shown that physical exercise reduces the excitability of neurons in the central nervous system, so there would be less sensitivity to pain [14,15]. Physical exercise has even been shown to prevent the development of chronic pain [15]. Previous research using Medical Expenditure Panel Survey (MEPS) data has shown a correlation between older adults (≥50 years) with pain who frequently exercise and lower medication costs [16].

There remains a gap in the literature about the factors associated with frequent physical exercise in US adults with pain. This knowledge may be useful to help create better-personalized interventions that include exercise in pain management protocols. Therefore, this study aimed to answer the question of which characteristics, including self-perceived pain severity, are associated with frequent physical exercise in US adults. Thus, the objective of this study was to identify the characteristics associated with frequent physical exercise in US adults.

## 2. Materials and Methods

### 2.1. Study Design and MEPS Data Source

This cross-sectional database study utilized the 2020 MEPS data. MEPS is conducted on behalf of the Agency for Healthcare Research and Quality, which is part of the US Department of Health and Human Services [17]. MEPS is a collection of large-scale surveys that collect data on individuals across the US. These variables include many demographic variables, economic and employment variables, insurance variables, limitation variables, access to healthcare variables, satisfaction with care variables, healthcare status variables, healthcare cost variables, and healthcare utilization variables. In 2020, data were collected for 27,805 people [18]. MEPS was selected as the data source for this study because it uses a complex survey design that can produce nationally representative estimates of the US civilian, non-institutionalized population when the appropriate weighting variable is employed in the analysis. The 2020 panel survey design involves multiple rounds, or panels, of interviews from a subsample of the 2019 National Health Interview Survey [18]. Data are typically collected five times over a two-year period and data for each year are later collated by MEPS staff. In 2020, due to the COVID-19 pandemic, additional data collection rounds were necessary [19]. Before data collection begins, MEPS is approved by an Institutional Review Board and all participants provide informed consent. MEPS has three components to the survey including: the household component, the insurance component, and the medical provider component [19]. This study utilized the MEPS household component. The MEPS household component collects data from all individuals in the households surveyed. Data in the household component are supplemented as available by MEPS staff from the insurance and medical provider components [19]. MEPS data are collated by MEPS staff and made publicly available for researchers to download from their websites [18].

### 2.2. Study Inclusion Criteria

To be included in this study, 2020 MEPS participants had to be alive for the full year (as determined by the person disposition variable), aged ≥18 years, and reported data for the frequent physical exercise question and the pain question in the MEPS survey. The frequent physical exercise and pain variables are described in greater detail below [19,20]. 

### 2.3. Dependent Variable

The dependent variable was frequent physical exercise. This variable was developed based on responses to an item in the Additional Healthcare Questions section of MEPS that asked if the person currently spends half an hour or more in moderate to vigorous physical activity at least five times a week. This item had yes or no response options for participants, which were also the response options used for analysis [19,20]. 

### 2.4. Independent Variable

The independent variable was pain severity. This variable was developed based on responses to the MEPS self-report item that asked: During the past four weeks how much did pain interfere with your normal work (including both work outside the home and housework)? This item had the following response options for participants: extreme, quite a bit, moderate, little, or none. For the purposes of this study, the same five levels of pain were used for analysis [19,20].

### 2.5. Control Variables

Control variables of relevance were identified in the dataset and organized into one of four categories for analysis (demographic, economic, limitation, or health variables). Demographic variables included age (≥65 years, 40–64 years, 18–39 years), sex (male, female), Hispanic (yes, no), white race (yes, no), and married (yes, no). Economic variables included more than high school education (yes, no), employed (yes, no), low income (yes, no), and health coverage (private, public, none). Limitation variables included activity of daily living (ADL) limitation (yes, no), instrumental activity of daily living (IADL) limitation (yes, no), functional limitation (yes, no), and work limitation (yes, no). Health variables included good mental health (yes, no), good general health (yes, no), multimorbidity (yes, no), smoker (yes, no), and overweight/obese (yes, no) [19,20]. A summary of the items asked and their response options in MEPS that were used in this analysis is provided in Appendix A.

### 2.6. Data Analysis

The characteristics of individuals who reported doing frequent physical exercise versus those who did not were compared using chi-squared tests. A univariate logistic regression model assessed the association between pain severity levels and frequent physical exercise status. A series of four multivariable logistic models assessed the association between pain severity levels and frequent physical exercise status, adjusting for: (1) demographic variables; (2) demographic and economic variables; (3) demographic, economic, and limitation variables; and (4) demographic, economic, limitation, and health variables. A significance level of 0.05 was selected a priori. Missing data were not included in the analysis. Analysis of data was conducted using SAS (SAS Institute Inc., Cary, NC, USA). This study was conducted and reported following the Strengthening the Reporting of Observational Studies in Epidemiology (STROBE) guidelines [21].

## 3. Results

**Figure 1:**
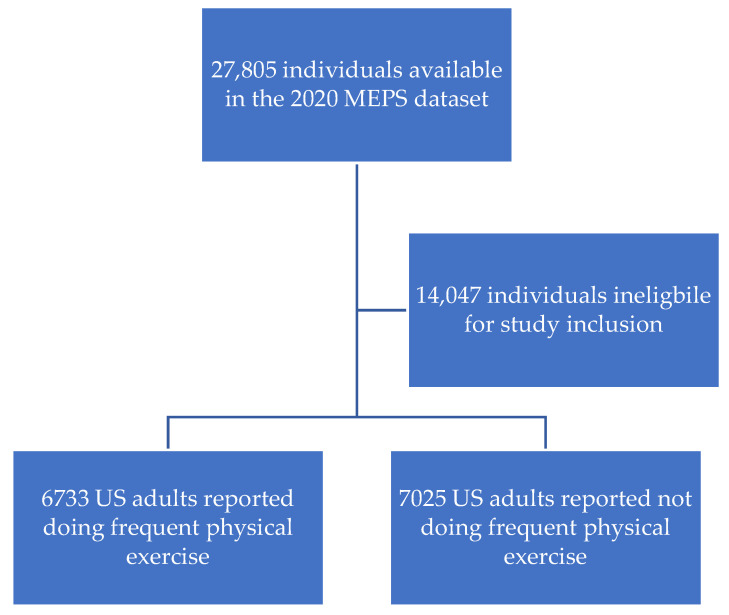
Participant Eligibility Flow Chart.

A total of 13,758 individuals from the dataset were included in the study. This represented a weighted sample of 240,629,358 individuals. Of these, 6733 reported doing frequent physical exercise, which represented a weighted sample of 121,151,908 (50.3%; 95% confidence interval [CI]: 48.9%, 51.8%). Meanwhile, 7025 reported not doing frequent physical exercise, which represented a weighted sample of 119,477,450 (49.7% (95% CI: 48.2%, 51.1%). See Figure 1.

Table 1 delineates the characteristics of individuals included in the study stratified by frequent physical exercise status. In total, most individuals did not have pain (62.9%). Of those who did have pain, the most common was little pain (22.6%), then moderate pain (7.2%), quite a bit of pain (5.3%), and extreme pain (2.0%). In total, the most common age group was 40–64 years (40.8%). The majority were female (51.7%), non-Hispanic (83.0%), white (78.3%), were married (51.8%), had more than high school education (59.9%), were employed (65.2%), did not have low income (75.1%), had private health coverage (68.1%), had no ADL limitations (98.4%), no IADL limitations (96.8%), no functional limitations (87.7%), and no work limitations (90.8%), had good mental health (90.7%), had good general health (88.0%), had no multimorbidity (58.3%), were non-smokers (88.1%), and were overweight/obese (66.2%). 

Table 2 presents the unadjusted associations between levels of pain severity and frequent physical exercise status among US adults. Compared to those with no pain, greater levels of pain severity were each associated with lower odds of reporting frequent physical exercise. That is, US adults with extreme pain had lower odds of reporting doing frequent physical exercise than US adults with quite a bit of pain, etc. 

Table 3 displays the adjusted associations between pain severity and frequent physical exercise among US adults. Greater levels of pain severity were associated with lower odds of reporting doing frequent physical exercise in all multivariable models except the final fully adjusted model, where only extreme pain and quite a bit of pain were associated with lower odds of reporting doing frequent physical exercise (i.e., moderate and little pain were no longer significantly associated). In the final fully adjusted model, Hispanic ethnicity (versus non-Hispanic ethnicity), having a functional limitation (versus not), and being overweight/obese (versus not) were all associated with lower odds of reporting doing frequent physical exercise. Conversely, ≥65- and 40–64-year-olds (versus 18–39-year-olds), males (versus females), white race (versus not white race), private or public health coverage (versus none), and good general health (versus poor general health) were all associated with greater odds of reporting doing frequent physical exercise. 

## 4. Discussion

This study reports the association between pain severity and frequent physical exercise (150 h a week of moderate to vigorous intensity) among US adults. The study also reports the factors in different categories (demographics, economic, limitation, and health) and their association with frequent physical exercise among this population. These findings are discussed in turn below.

In the univariate analysis (Table 2), we observed that greater amounts of pain experienced by an individual were correlated with lower odds of reporting frequent physical exercise. We observed similar results in the early multivariate analysis models (Table 3), although there was some minor variation in the effect size. However, in the final adjusted model (after adjusting for demographic, economic, limitation, and health variables), there was a significant correlation between extreme pain and quite a bit of pain with frequent physical exercise, but no correlation between moderate or little pain and frequent physical exercise. This finding is supported by previous studies that showed the amount of physical activity performed decreases based on the frequency and intensity of pain [22,23]. Ray et al. found those who reported pain had lower odds of participating in physical activity, and meeting the guidelines for physical activity was associated with lower odds of reporting pain [22]. Zadro et al. found that twins who experience lower back pain have lower odds of meeting the World Health Organization physical activity guidelines than those who do not experience lower back pain [23]. Another study tracking sedentary behavior in older US adults found that those who were more sedentary were more likely to report experiencing some amount of pain [24]. These findings should be considered when adding frequent physical exercise to pain management plans for adults experiencing pain because patients who are experiencing extreme pain are the least likely to be exercising compared to those experiencing little pain.

Of the demographic variables that were analyzed, age, sex, ethnicity, and race were associated with frequent physical activity. Adults aged ≥65 years and 40–64 years had higher odds of reporting frequent physical exercise than adults aged 18–39. The literature shows several studies of older adults, however, there are knowledge gaps for adults aged 18–64 that this study can fill. A global study showed that a quarter of adults of all ages did not meet the physical activity guidelines set in 2018 by the World Health Organization in [25]. Another study that supports this finding analyzed the association between electronic wearable devices and meeting US physical activity guidelines and shows that adults aged ≥65 years and 50–64 years have higher odds of meeting the guidelines than 18–49-year-olds [26]. These findings could be due to older adults in retirement who have more time for leisure physical activity, or they are trying to maintain their health more than younger adults. 

In the current study, males were associated with higher odds of reporting frequent physical exercise than females. Globally adult men are more active than women, at 76.6% and 68.3%, respectively. This gap begins early with young girls (3–11 years) experiencing less enjoyment of physical activity than boys [25]. Another study showed that 28.3% of men and 20.4% of women in 2020 met all the US guidelines for physical activity [27]. Our analysis showed that people who identify as Hispanic had lower odds of reporting frequent physical exercise than people who identify as non-Hispanic and that people who identify as White had higher odds of reporting frequent physical exercise compared to those who do not identify as White. These findings are similar to those reported by a study using the American Time Use Surveys that found people who identify as Black, Asian, or Hispanic have a lower probability of performing leisurely physical activity than those who identify as non-Hispanic White [28]. Specifically, people who identify as Hispanic have reported the highest rate (31.7%) of inactivity in the US as of 2020, which could largely be related to socioeconomic status [29]. Clinicians should consider these results when creating care plans for people experiencing pain, because different demographics may be more likely to do frequent physical exercise than others.

The only economic factor associated with self-reported frequent physical exercise in the current study was health coverage. People who had health insurance coverage, both private and public, were associated with higher odds of reporting frequent physical exercise than people who do not have health insurance. The reasons to explain this are unclear, but we offer some potential explanations. It may be that those with insurance are more able to afford the time to exercise, purchase gym memberships, or live in an area with safe access to outdoor recreation space due to their higher socioeconomic status. It may also be that those with insurance are more invested in protecting their health through frequent physical exercise than those with no insurance (e.g., a sort of healthy user bias). In addition, those with insurance have access to healthcare professionals who can offer advice on exercise for the patient to act upon [30]. More novel explanations include private health insurance companies offering incentives for individuals to use physical activity trackers and sending the monitored data back to the insurance company, with the goal of increasing frequent physical exercise, decreasing the prevalence of chronic disease, and reducing healthcare expenditures [31]. Health insurance creating incentives for their customers to exercise could be a great way to promote frequent physical exercise in US adults and increase health outcomes.

Among the limitation variables, people who reported having a functional limitation were associated with lower odds of reporting frequent physical exercise than those without a functional limitation. However, none of the other limitation variables showed a significant association with frequent physical exercise. This may be because of the known association between decreased physical activity and functional decline for day-to-day tasks [32]. Depending on the directionality, which this study cannot ascertain, greater efforts are needed to promote frequent physical exercise to prevent functional decline, and interventions to prevent or improve functional status are needed to facilitate improvement in frequent physical exercise status. 

Of the health variables, obesity and good mental health were associated with frequent physical exercise. Those who were overweight had lower odds of reporting frequent physical exercise. This is perhaps unsurprising given the known relationship between obesity and frequent physical exercise [33]. Obesity is known to increase physical limitations and negatively influence musculoskeletal health [34]. However, another study found most of the obese subjects were physically active so this factor may depend on what population is being analyzed, as well as the definitions of physical exercise used, e.g., any physical exercise versus the definition used in the current study [35]. This finding could be helpful in adding to the campaigns against obesity; while there are many negative health effects of obesity, people may not consider the association between obesity and pain. Good general health was associated with higher odds of reporting frequent physical exercise than those who are not in good general health. Healthy behaviors, such as frequent physical exercise, are associated with the benefits of an overall healthy lifestyle [36]. People who experience the benefits of frequent physical exercise may be influenced to keep exercising and maintaining a healthy lifestyle, promoting a positive feedback loop. The importance of good general health shows that there is a need for policies that implement wellness programs from insurance companies or employers. 

This study suggests the importance of frequent physical exercise for those experiencing some degree of pain. In clinical practice, it may be appropriate for healthcare providers to advise moderate physical exercise to people experiencing pain, or even as a preventative measure to stop the manifestation of chronic pain. From a policy perspective, the findings from this study lend evidence to the need to create policies that encourage the importance of frequent physical exercise. This could exhibit itself as insurance or employer’s wellness programs giving incentives to exercise, or teaching children that physical exercise is important to maintain health and prevent any ailments. If policies such as these were to be enforced there may be an increase in US adults practicing frequent physical exercise and that is associated with decreased odds of experiencing pain. From a research perspective, in the future, it would be interesting to compare the effectiveness of consistent physical exercise as a pain management strategy, particularly among US adults.

This study is susceptible to limitations. Specifically due to the nature of the study design, there is a lack of a causal relationship. Reporting bias may have also taken place, as this was a self-reported survey, and the participants may overestimate the frequency and intensity of their physical exercise. Recall bias should also be considered as the MEPS was conducted over a two-year period; thus, people may have inaccurately recalled how often they were exercising and at what intensity. Finally, several of the variables were dichotomous in nature which possibly could have resulted in less detailed information than continuous data. Some variables, such as type and location of pain, were not available in the dataset. Future research could use a different dataset to examine subgroups of this population to determine if there are differences in the type and location of pain.

## 5. Conclusions

This cross-sectional database study of 13,758 US adults, weighted to represent approximately 240,629,358 US adults, identified extreme pain and quite a bit of pain (relative to no pain) were associated with lower odds of reporting frequent physical exercise (half hour or more of moderate to vigorous physical activity at least five times a week). In addition, several demographic, economic, limitation, and health variables were also associated with frequent physical exercise, including age, sex, ethnicity, race, health coverage, functional limitations, general health, and weight. Future studies are warranted to establish a cause-and-effect relationship between pain and physical exercise among US adults.

## Figures and Tables

**Table 1:** Characteristics of the study population.

	Frequent Physical Exercise: Yes N = 6733	Frequent Physical Exercise: No N = 7025	Total N = 13,758	*p* Value
	Percent [95% CI]	Percent [95% CI]	Percent [95% CI]	
Pain:				<0.0001
Extreme	0.7 [0.4, 0.9]	3.3 [2.9, 3.8]	2.0 [1.7, 2.2]	
Quite a bit	3.6 [2.9, 4.2]	7.1 [6.4, 7.8]	5.3 [4.9, 5.8]	
Moderate	6.2 [5.5, 6.9]	8.2 [7.4, 9.0]	7.2 [6.7, 7.7]	
Little	22.3 [21.1, 23.6]	22.8 [21.5, 24.0]	22.6 [21.6, 23.5]	
None	67.2 [65.7, 68.8]	58.6 [57.0, 60.1]	62.9 [61.8, 64.1]	
Demographic variables:				
Age				0.5548
≥65	21.8 [20.5, 23.1]	22.7 [21.4, 24.0]	22.3 [21.2, 23.3]	
40–64	40.8 [39.3, 42.4]	40.8 [39.2, 42.3]	40.8 [39.6, 42.0]	
18–39	37.4 [35.7, 39.1]	36.5 [34.8, 38.2]	36.9 [35.7, 38.2]	
Sex				<0.0001
Male	52.3 [51.0, 53.6]	44.1 [42.8, 45.5]	48.3 [47.4, 49.1]	
Female	47.7 [46.4, 49.0]	55.9 [54.5, 57.2]	51.7 [50.9, 52.6]	
Hispanic				<0.0001
Yes	15.0 [13.0, 17.0]	19.0 [16.6, 21.4]	17.0 [15.0, 19.0]	
No	85.0 [83.0, 87.0]	81.0 [78.6, 83.4]	83.0 [81.0, 85.0]	
White race				<0.0001
Yes	80.9 [79.0, 82.7]	75.6 [73.5, 77.8]	78.3 [76.6, 80.0]	
No	19.1 [17.3, 21.0]	24.4 [22.2, 26.5]	21.7 [20.0, 23.4]	
Married				0.0016
Yes	53.3 [51.5, 55.2]	50.3 [48.7, 51.8]	51.8 [50.4, 53.2]	
No	46.7 [44.8, 48.5]	49.7 [48.2, 51.3]	48.2 [46.8, 49.6]	
Economic variables:				
More than high school education				0.0001
Yes	62.1 [60.2, 64.1]	57.7 [55.6, 59.8]	59.9 [58.3, 61.6]	
No	37.9 [35.9, 39.8]	42.3 [40.2, 44.4]	40.1 [38.4, 41.7]	
Employed				<0.0001
Yes	67.9 [66.3, 69.5]	62.4 [60.8, 64.1]	65.2 [64.0, 66.4]	
No	32.1 [30.5, 33.7]	37.6 [35.9, 39.2]	34.8 [33.6, 36.0]	
Low income				<0.0001
Yes	21.1 [19.5, 22.7]	28.7 [26.7, 30.6]	24.9 [23.3, 26.4]	
No	78.9 [77.3, 80.5]	71.3 [69.4, 73.3]	75.1 [73.6, 76.7]	
Health coverage				<0.0001
Private	71.3 [69.4, 73.2]	64.8 [63.1, 66.4]	68.1 [66.6, 69.5]	
Public	22.1 [20.5, 23.7]	26.4 [25.0, 27.9]	24.3 [23.1, 25.4]	
None	6.6 [5.6, 7.6]	8.8 [7.5, 10.0]	7.7 [6.7, 8.6]	
Limitation variables:				
ADL limitation				<0.0001
Yes	0.7 [0.5, 1.0]	2.5 [2.0, 3.0]	1.6 [1.3, 1.9]	
No	99.3 [99.0, 99.5]	97.5 [97.0, 98.0]	98.4 [98.1, 98.7]	
IADL limitation				<0.0001
Yes	1.8 [1.3, 2.3]	4.5 [3.8, 5.2]	3.2 [2.7, 3.6]	
No	98.2 [97.7, 98.7]	95.5 [94.8, 96.2]	96.8 [96.4, 97.3]	
Functional limitation				<0.0001
Yes	7.4 [6.7, 8.2]	17.2 [15.9, 18.5]	12.3 [11.5, 13.1]	
No	92.6 [91.8, 93.3]	82.8 [81.5, 84.1]	87.7 [86.9, 88.5]	
Work limitation				<0.0001
Yes	5.7 [4.9, 6.5]	12.7 [11.6, 13.9]	9.2 [8.4, 10.0]	
No	94.3 [93.5, 95.1]	87.3 [86.1, 88.4]	90.8 [90.0, 91.6]	
Health variables:				
Good mental health				<0.0001
Yes	93.3 [92.5, 94.2]	88.0 [87.0, 89.1]	90.7 [90.0, 91.4]	
No	6.7 [5.8, 7.5]	12.0 [10.9, 13.0]	9.3 [8.6, 10.0]	
Good general health				<0.0001
Yes	92.7 [91.8, 93.7]	83.3 [82.2, 84.3]	88.0 [87.2, 88.8]	
No	7.3 [6.3, 8.2]	16.7 [15.7, 17.8]	12.0 [11.2, 12.8]	
Multimorbidity				<0.0001
Yes	38.4 [36.8, 40.0]	45.1 [43.5, 46.8]	41.7 [40.6, 42.9]	
No	61.6 [60.0, 63.2]	54.9 [53.2, 56.5]	58.3 [57.1, 59.4]	
Smoker				0.0620
Yes	11.2 [10.0, 12.4]	12.7 [11.6, 13.8]	11.9 [11.1, 12.8]	
No	88.8 [87.6, 90.0]	87.3 [86.2, 88.4]	88.1 [87.2, 88.9]	
Overweight/obese				<0.0001
Yes	61.6 [59.8, 63.5]	71.0 [69.3, 72.7]	66.2 [64.8, 67.6]	
No	38.4 [36.5, 40.2]	29.0 [27.3, 30.7]	33.8 [32.4, 35.2]	

CI = confidence interval; ADL = activities of daily living; IADL = instrumental activities of daily living. Differences between groups were compared using chi-square tests.

**Table 2:** Univariate association between pain severity and frequent physical exercise status among United States adults.

Pain	Odds Ratio [95% CI]
Extreme vs. none	**0.2 [0.1, 0.2]**
Quite a bit vs. none	**0.4 [0.4, 0.6]**
Moderate vs. none	**0.7 [0.6, 0.8]**
Little vs. none	**0.9 [0.8, 0.9]**

CI = confidence interval. Statistically significant results are indicated in **bold** font.

**Table 3:** Multivariate associations between pain severity and frequent physical exercise status among United States adults.

	Adjusted for Pain & Demographic Variables	Adjusted for Pain, Demographic & Economic Variables	Adjusted for Pain, Demographic, Economic, & Limitation Variables	Adjusted for Pain, Demographic, Economic, Limitation, & Health Variables
	Odds Ratio [95% CI]	Odds Ratio [95% CI]	Odds Ratio [95% CI]	Odds Ratio [95% CI]
Pain:				
Pain, extreme vs. none	**0.2 [0.1, 0.2]**	**0.2 [0.1, 0.3]**	**0.3 [0.2, 0.5]**	**0.4 [0.2, 0.7]**
Pain, quite a bit vs. none	**0.4 [0.3, 0.5]**	**0.4 [0.4, 0.6]**	**0.5 [0.4, 0.7]**	**0.7 [0.5, 0.9]**
Pain, moderate vs. none	**0.6 [0.5, 0.7]**	**0.6 [0.5, 0.7]**	**0.7 [0.6, 0.9]**	0.8 [0.7, 1.1]
Pain, little vs. none	**0.8 [0.7, 0.9]**	**0.8 [0.7, 0.9]**	**0.8 [0.7, 1.0]**	0.9 [0.8, 1.0]
Demographic variables:				
Age, ≥65 vs. 18–39	1.1 [0.9, 1.2]	1.1 [0.9, 1.2]	1.2 [1.0, 1.5]	**1.3 [1.1, 1.6]**
Age, 40–64 vs. 18–39	1.1 [0.9, 1.2]	1.0 [0.9, 1.2]	1.2 [1.0, 1.3]	**1.2 [1.1, 1.4]**
Sex, male vs. female	**1.4 [1.3, 1.5]**	**1.4 [1.3, 1.5]**	**1.3 [1.2, 1.4]**	**1.4 [1.2, 1.5]**
Hispanic, yes vs. no	**0.7 [0.6, 0.8]**	**0.7 [0.6, 0.8]**	**0.7 [0.6, 0.8]**	**0.7 [0.6, 0.9]**
White race, yes vs. no	**1.5 [1.3, 1.7]**	**1.4 [1.2, 1.6]**	**1.5 [1.3, 1.8]**	**1.5 [1.3, 1.8]**
Married, yes vs. no	1.0 [1.0, 1.1]	1.0 [0.9, 1.1]	1.0 [0.9, 1.1]	1.0 [0.9, 1.1]
Economic variables:				
More than high school education, yes vs. no		1.0 [0.9, 1.1]	1.0 [0.9, 1.1]	1.0 [0.8, 1.1]
Employed, yes vs. no		1.0 [0.9, 1.2]	0.9 [0.8, 1.1]	1.0 [0.8, 1.1]
Low income, yes vs. no		**0.8 [0.7, 0.9]**	0.9 [0.7, 1.0]	0.9 [0.8, 1.1]
Health coverage, private vs. none		**1.3 [1.1, 1.6]**	**1.3 [1.0, 1.6]**	**1.3 [1.0, 1.6]**
Health coverage, public vs. none		**1.3 [1.1, 1.6]**	**1.3 [1.0, 1.7]**	**1.3 [1.0, 1.8]**
Limitation variables:				
ADL limitation, yes vs. no			0.7 [0.4, 1.3]	0.7 [0.4, 1.3]
IADL limitation, yes vs. no			0.9 [0.6, 1.4]	0.9 [0.6, 1.4]
Functional limitation, yes vs. no			**0.6 [0.5, 0.7]**	**0.6 [0.5, 0.7]**
Work limitation, yes vs. no			0.8 [0.7, 1.1]	0.9 [0.7, 1.2]
Health variables:				
Good mental health, yes vs. no				1.2 [0.9, 1.5]
Good general health, yes vs. no				**1.6 [1.3, 2.0]**
Multimorbidity, yes vs. no				0.9 [0.8, 1.0]
Smoker, yes vs. no				1.0 [0.8, 1.1]
Overweight/obese, yes vs. no				**0.7 [0.6, 0.8]**

CI = confidence interval. ADL = activities of daily living; IADL = instrumental activities of daily living. Statistically significant results indicated in **bold** font.

## Data Availability

Data are available from the corresponding author upon reasonable request.

## Appendix A. Summary of the Items Asked and Their Response Options in MEPS That Were Used in This Analysis

**Item**	**Response Options**
Moderate/vigorous physical exercise 5x a week	Yes, no
Pain limits normal work	Not at all, a little bit, moderately, quite a bit, extremely
Age as of 12/31/20	0–85 (top-coded)
Sex	Male, female
Hispanic ethnicity	Hispanic, not Hispanic
Race	White-no other race reported, black-no other race reported, American Indian/Alaska native-no other race, Asian/native Hawaiian/pacific islander-no other race reported, multiple races reported
Marital status-12/31/20	Married, widowed, divorced, separated, never married, under age 16-inapplicable
Years of Educ when first entered MEPS	No school/kindergarten only, elementary grades 1–8, high school grades 9–11, grade 12, 1 year college, 2 years college, 3 years college, 4 years college, 5+ years college
Employment status	Employed during the round, job to return to during the round, job during the round, not employed during the round
Family income as % of the poverty line	Poor/negative, near poor, low income, middle income, high income
Health insurance coverage indicator 2020	Any private, public-only, uninsured
ADL screener	Yes, no
IADL screener	Yes, no
Limitations in physical functioning	Yes, no
Any limitation on work/housework/schoolwork	Yes, no
Perceived mental health status	Excellent, very good, good, fair, poor
Perceived health status	Excellent, very good, good, fair, poor
Multiple diagnoses of high blood press	Yes, no
Coronary heart disease diagnosis	Yes, no
Angina diagnosis	Yes, no
Heart attack (MI) diagnosis	Yes, no
Other heart disease diagnoses	Yes, no
Stroke diagnosis	Yes, no
Emphysema diagnosis	Yes, no
Chronic bronchitis last 12 months	Yes, no
High cholesterol diagnosis	Yes, no
Cancer diagnosis	Yes, no
Diabetes diagnosis	Yes, no
Joint pain last 12 months	Yes, no
Arthritis diagnosis	Yes, no
Asthma diagnosis	Yes, no
How often smoke cigarettes	Every day, some days, not at all
Adult body mass index	Continuous
